# Small RNAs: Promising Molecules to Tackle Climate Change Impacts in Coffee Production

**DOI:** 10.3390/plants12203531

**Published:** 2023-10-11

**Authors:** Kellen Kauanne Pimenta de Oliveira, Raphael Ricon de Oliveira, Antonio Chalfun-Junior

**Affiliations:** Laboratory of Plant Molecular Physiology, Plant Physiology Sector, Institute of Biology, Federal University of Lavras, Lavras 3037, Brazil; kauannepimenta@gmail.com (K.K.P.d.O.); rapharicon@gmail.com (R.R.d.O.)

**Keywords:** sRNAs, *Coffea arabica* L., transcriptional regulation, crop breeding

## Abstract

Over the centuries, human society has evolved based on the ability to select and use more adapted species for food supply, which means making plant species tastier and more productive in particular environmental conditions. However, nowadays, this scenario is highly threatened by climate change, especially by the changes in temperature and greenhouse gasses that directly affect photosynthesis, which highlights the need for strategic studies aiming at crop breeding and guaranteeing food security. This is especially worrying for crops with complex phenology, genomes with low variability, and the ones that support a large production chain, such as *Coffea* sp. L. In this context, recent advances shed some light on the genome function and transcriptional control, revealing small RNAs (sRNAs) that are responsible for environmental cues and could provide variability through gene expression regulation. Basically, sRNAs are responsive to environmental changes and act on the transcriptional and post-transcriptional gene silencing pathways that regulate gene expression and, consequently, biological processes. Here, we first discuss the predicted impact of climate changes on coffee plants and coffee chain production and then the role of sRNAs in response to environmental changes, especially temperature, in different species, together with their potential as tools for genetic improvement. Very few studies in coffee explored the relationship between sRNAs and environmental cues; thus, this review contributes to understanding coffee development in the face of climate change and towards new strategies of crop breeding.

## 1. Introduction

Due to climate changes and the importance of crops to human life, a lot of studies have been carried out toward understanding plant plasticity and the development of more tolerant varieties in biotic and abiotic stresses, together with higher productivity [1,2,3,4,5]. One of these important crops is *Coffea* sp L., which presents a complex phenological cycle and whose coffee fruit production is mainly affected by temperature changes, rainfall, and altitude [6,7,8,9]. Climate projections indicate that by the end of this century, global temperature may increase by 0.2 to 1.0 °C in the best scenario and from 2.4 to 4.8 °C in the most pessimistic scenario in comparison to the period from 1986 to 2005 [10]. In the long term, climate change, combined with such phenomena as longer and more unpredictable droughts or excessive rainfall, threatens the sustainability of coffee production on a global scale [11,12], which is also expected for other important crops [13,14].

The *Coffea* genus belongs to the Rubiaceae family with 130 described species [15]. *Coffea arabica* (Arabica coffee) and *Coffea canephora* are responsible for almost all the coffee bean production in the world [16]. Interestingly, the vast majority of coffee species are diploid and allogamous, while *C. arabica* is an allotetraploid and autogamous species that originated from the natural hybridization of non-reduced gametes of *C. eugenioides* and *C. canephora* [17,18]. Furthermore, *C. arabica* has a narrow genetic base, as described in the topic below, and due to favorable climatic conditions, together with genetic improvement programs, Brazil has become its largest producer in the world [19]. In this way, it is clear that a need arises for intelligent strategies to promote diversity and support genetic improvement, guaranteeing coffee production and, in a broader aspect, the adaptation of crops to climate change. In other species, one of these strategies includes the identification of new regulatory molecules such as small RNAs (sRNAs) [20,21,22]. These small RNAs act as post-transcriptional regulators of gene expression, degrading or blocking the target mRNA and regulating different processes, such as development, differentiation, flowering, metabolism, response to abiotic stress, and plant defense [23,24,25].

Elevated temperatures and heat stress negatively affect the distribution and yield of commercially important plants worldwide [26,27,28]. When the temperature rises 5 °C or more above the ideal, which is specific for each species, it is experienced as heat stress by all living organisms [29]. In the coffee tree, at the beginning of flowering, high temperatures associated with the intense water deficit cause the death of the pollen tubes by dehydration, causing the abortion of the flowers [30] or premature opening of the floral buds [31], which ends up generating losses in production. Furthermore, continuous exposure of *C. arabica* to temperatures close to 30 °C or more can result not only in repressed growth but also in anomalies such as yellowing of leaves [32], growth of tumors at the base of the stem [33], changes in the enzymes of the photosynthetic apparatus [34], and worsening of the grain quality [35].

Some studies have already been carried out in which microRNAs (miRNAs), a class of sRNAs responsive to heat stress by high temperatures, have been characterized, such as in *Populus tomentosa* [36], *Arabidopsis thaliana* [29], *Oryza sativa* [37], *Saccharina japonica* [38]; *Solanum lycopersicum* [39], *Gossypium hirsutum* [40], and *Musa* spp. AAA [41]. However, despite some miRNAs in common, the list of miRNAs with altered expression varies with the degree of stress and between species. Although sRNAs in coffee plants have already been addressed in some studies, such as the identification of miRNAs and their biogenesis pathways [42,43,44,45], miRNAs in floral development [46] and reproductive development [47], there is still a gap relating the role of sRNAs in temperature control in these species. Therefore, considering the peculiar aspect of *C. arabica* origin related to the narrow diversity and the need for more adapted genotypes in the face of climate changes, this review contributes to the understanding of the roles of sRNAs in coffee plants and discusses perspectives on its use as tools for crop breeding.

## 2. Coffee Plant: Origin, Phenology, and Environment-Related Aspects

In order to understand how the coffee tree behaves in the face of climate change, it is important to know how this plant emerged and spread throughout the world and how its very peculiar phenological aspects can contribute to this. The coffee tree belongs to the Rubiaceae family, genus *Coffea*, with 130 described species [15], the vast majority being diploid and allogamous; however, only *C. arabica* (Arabica coffee) and *C. canephora* (Robusta/Conilon coffee) are cultivated expressively. The other two species are cultivated on a smaller scale, *C. liberica* (Liberica coffee) and *C. dewevrei* (Excelsa coffee) [48]. In Brazil, 80% of the total production is *C. arabica* [16].

The origin and center of genetic diversity of *C. arabica* is in Southwestern Ethiopia [49,50], while *C. canephora* originated in Central and Western Africa [51,52,53]. *C. arabica* originated from the natural hybridization of non-reduced gametes of the diploid species, *C. eugenioides* and *C. canephora* [17,18,54], forming an allotetraploid species, 2n = 4x = 44, and it is considered autogamous with about 10% of allogamy [55]. This very rare hybridization event was estimated to have occurred from a single plant around 20,000 years ago [18] or, considering other predictions, between 100,000 and 665,000 years ago [56,57]. Particular events of that period seem to be related to thermal shocks that promoted the fusion of gametes and the shutdown of self-incompatibility systems of parent species, allowing for autogamy and reproduction [18], which can be related to the insertion of retrotransposons [56]. Transposons are endogenous DNA sequences capable of moving and multiplying in the host genome, impairing genes but also contributing to genome evolution [58,59,60], and in agreement with the stated before, these elements present higher activities under temperature changes [61,62]. Although the genetic base of *C. arabica* is narrow, the species has cultivars with great variability due to factors such as crossings, mutations, agronomic practices, and environment [63,64]. Currently, this species is widely distributed, being cultivated in regions with higher altitudes and milder temperatures, between 18 °C and 21 °C, in the American and Asian continents, as well as in some regions of Africa [64].

On the other hand, *C. canephora* is a diploid species, with 2n = 22 chromosomes, allogamous, originating from a wide hot, humid, and low-altitude region of the African continent, which extends from Guinea to the Democratic Republic of Congo [17,65]. *C. canephora* is widely cultivated in the African, American, and Asian continents, in places of low altitude and higher temperatures, with an annual average between 22 °C and 26 °C [66]. In regions with higher temperatures and abundant humidity, plants of the *C. canephora* species can reach up to 5 m in height. They are usually multi-stemmed, even in commercial crops with frequent thinning [64]. It is a highly polymorphic species with extensive geographical distribution, showing great adaptability to different environmental conditions [64]. This species was introduced in Brazil around 1920, in Espírito Santo, and its varieties have great variability in relation to the different agronomic and morphological characteristics due to the origin of the species and the fact that it reproduces by cross-self-fertilization [67].

Regarding its phenology, contrary to most angiosperms, which emit inflorescences and bear fruit in the same phenological year, coffee needs two years to complete its cycle [30]. The first year begins with vegetative growth characterized by the development of plagiotropic branches from orthotropic branches. The first phase, which runs from September to March, begins with the formation of axillary buds at the nodes of the primary plagiotropic branches [30,68]. From January to July, the differentiation of floral buds between early and late cultivars of *C. arabica* begins; it is known that the quality and intensity of light, as well as the photoperiod, affect plant growth, directly influencing floral development and many other characteristics; however, the inductive stimulus of the coffee reproductive cycle is still unclear and may be affected by the interaction of different environmental factors, in which molecular aspects are little explored [68]. Recently, López et al. [68] reviewed the endogenous and environmental factors related to the flowering process of *C. arabica* and proposed a model for the induction and floral development in Brazilian environmental conditions, where the floral induction starts in January, extending until July for early and late cultivars. However, based on gene expression data, López et al. [69] suggest a continuum of floral induction that allows different starting points for floral activation, which explains developmental asynchronicity and prolonged anthesis events in tropical perennial species.

## 3. Impacts of Increased Temperature and CO_2_ on Coffee Growing

Drought and unfavorable temperatures have always been described as the main climatic limitations for coffee production [70]. The main effects of climate change are displacement of optimal growing zones, changes in precipitation (amount and distribution), changes in crop disease/pest dynamics, and loss of agricultural land due to sea level rise and/or desertification [71]. As changes in the global climate are recognized, and coffee cultivation spreads to different regions where it was not cultivated before, concerns are increasing about these factors and the possible consequences for the production of this crop [32,70,71,72,73].

Indeed, temperature can limit the economic exploitation of coffee, in part because coffee growth is particularly affected by high and low temperatures [74]. The optimum average temperature range for *C. arabica* is reported to be between 18 and 21 °C; however, it should be noted that cultivars selected under intensive management conditions have allowed *C. arabica* plantations to be spread to regions with average annual temperatures of up to 24–25 °C, with satisfactory yields [35,73,75,76,77].

On the other hand, in regions with an average annual temperature below 18 °C [13,74,78], growth is largely repressed; in any case, it is clear that large variations in temperature cause defects in the beans, modify their biochemical composition, and alter the final quality of the beverage [13,74,78]. However, the pessimistic data obtained so far on how climate change affects the coffee crop had not considered the potential positive effects of high atmospheric CO_2_ concentration on coffee plant photosynthesis or the role of CO_2_ on heat tolerance and recognized resilience of elite coffee genotypes to acclimatize to stressful conditions [31].

In the physiological aspect, in general, plants feel and respond directly to the increase in atmospheric CO_2_ concentration through an increase in the rate of net photosynthesis (*A*) and reduction in stomatal conductance (*g_s_*); this is the basis for the effect of CO_2_ fertilization with a corresponding increase in yields [79,80]. Increases in *A* and CO_2_ concentration in the chloroplast of C3 plants are associated with stimulation of the rate of carboxylation of ribulose-1,5-bisphosphate carboxylase/oxygenase (RuBisCO) [80]. Furthermore, it has been shown systematically, but not universally, that *g_s_* decreases with high levels of CO_2_ concentration [81]. In general, decreases in *g_s_* often lead to lower transpiration rates and greater water use efficiency. However, this also results in latent heat loss and increasing leaf temperatures, which may impair crop performance in a global warming scenario [31].

Regarding productivity, the beneficial effect of increasing CO_2_ was demonstrated in a study carried out with *C. arabica* plants monitored for 3 years, where a higher yield of grain production was verified than in plants grown under ambient CO_2_ concentration [82]. However, the effects of the combination of high temperatures and CO_2_ concentrations on production yield and grain quality still need to be better explored.

In a study of *C. arabica* and *C. canephora* plants cultivated for 10 months at 25/20 °C (day/night) under 380 and 700 μL of CO_2_ L^−1^ and then subjected to an increase in temperature (0.5 °C per day) up to 42/34 °C (day/night), higher concentrations of CO_2_ strongly contributed to the impact of temperature on both species, promoting greater efficiency in water use, not causing negative photosynthetic regulation, in addition to increasing the protection molecules, as well as the activity of some antioxidant enzymes [76]. High concentrations of CO_2_, therefore, play a role in the sustainability of the coffee crop under future scenarios of climate change [75,76], as demonstrated by recent works on strengthening the capacity of moderate and severe drought tolerance [83,84,85,86].

Thus, although the studies are focused on a better understanding of how the physiological aspects respond to climatic variations and the importance of CO_2_ as a mitigator of the damage caused by the increase in temperature, crucial issues still need to be elucidated, such as, for example, how the concentration increased CO_2_ levels together with increased temperature can affect the plant–pest/disease balance, abnormal flower formation, and fruit development and quality. In addition, associating these results with molecular and genetic data may help to develop new ways to improve coffee, and in this sense, exploring sRNAs, in general, can be a good way to fill this gap since they are fine regulators of gene expression. 

## 4. How Do Genetic and Molecular Components Contribute to the Coffee Plant’s Physiological Responses to Climate Change?

Genetic and molecular aspects are crucial to determining how organisms respond to different environmental conditions; however, studies relating these factors to physiological responses are limited. In the genetic and molecular aspect, the adaptive and evolutionary capacities of *C. arabica* and its parents (*C. eugenioides* and *C. canephora*) were evaluated under four thermal regimes (RTs; 18–14 °C, 23–19 °C, 28–24 °C, and 33–29 °C) [87]. The data indicate that *C. arabica* activates the genome inherited from each parent for different temperature conditions. For example, the growth rate was similar to that of *C. canephora* under the hottest RT and that of *C. eugenioides* under the coldest RT. For metabolite contents, *C. arabica* showed a response similar to that of *C. canephora* in the hottest RT [87].

At the level of gene expression, few differences between the allopolyploid and its parents were observed for studied genes linked to photosynthesis, respiration, and the circadian clock, while genes linked to redox activity showed a greater capacity of the allopolyploid for homeostasis [87]. In this way, the global transcriptional response to RTs of *C. arabica* was more homeostatic compared to its parents, and this provided greater phenotypic homeostasis when faced with unsuitable environments for the diploid parent species [87].

In another study, two genotypes of *C. arabica* (Acauã and Catuaí) were compared regarding leaf physiology, transcriptome, and carbohydrate/protein levels under optimal and elevated temperatures. It was demonstrated that the Acauã cultivar had lower leaf temperatures in both conditions than cv. Catuaí, while little or no difference was observed for the other leaf physiological parameters [88]. However, the genotypes showed a transcriptional restriction in the warmer temperatures. Differentially expressed genes responsive to temperature elevation revealed shared and genotype-specific genes, mainly related to carbohydrate metabolism [88]. These findings revealed intraspecific differences in the interconnected transcriptional and metabolic pathways that respond to warmer temperatures, potentially linked to coffee thermotolerance [88].

## 5. The sRNA Characteristics and Their Roles in Plant Stress Mediation

Most of the transcribed sequences of a genome do not encode proteins and are called non-coding RNAs (ncRNAs). Among the non-coding RNAs, we highlight three major groups of small RNAs (sRNAs) that act repressing gene expression through the transcriptional and post-transcriptional gene silencing pathways, TGS and PTGS, respectively, miRNA, small interfering RNAs (siRNAs), and phased secondary small interfering RNAs (phasiRNAs) [23,89,90]. Although sRNAs are biochemically very close, they differ from each other in their biogenesis to their mode of action. For example, microRNAs (miRNAs) are transcribed from MIR genes, while siRNAs are cleavage products of other RNAs [23,40,91], and phasiRNAs constitute an important category of small RNAs in plants, but most of their functions are still poorly defined [90].

MiRNAs are an extensive class of endogenous, small, non-coding RNAs (sncRNAs), ranging from 20 to 24 nucleotides in length, which are involved in mediating and silencing post-transcriptional gene expression. Although inhibition of translation by cleavage is a process classically mediated by siRNAs, miRNAs also play this role [23,92]. MiRNAs are encoded by MIR, which are transcribed by RNA polymerase II (POL II), while sRNAs are generally transcribed from the action of polymerases IV and V exclusive to plants [93]. The primary miRNA transcripts (pri-miRNA) are 5′-encapsulated and polyadenylated and are capable of forming imperfect folding structures called “hairpins”, which have a 5p and a 3p arm, with a size similar to the transcripts that encode proteins [92,94].

Although MIR genes often encode a single RNA transcript, there are few documented polycistronic miRNAs in plants, a phenomenon that is much more common in animals. Transcription of some miRNA loci can generate transcripts with different hairpin structures and sequences, but which can still be processed by the miRNA biogenesis machinery, resulting in different “mature” sequences [23,92,94]. In plants, the primary transcript (pri-miRNA) is stabilized by DAWDLE-binding proteins (DDL) and is further processed in the nucleus by the endonuclease activity of DICER-LIKE1 (DCL1) with the help of other proteins, such as RNA-binding proteins double-stranded (dsRNA) HYPONASTIC LEAVES 1 (HYL1) and SERRATE (SE) in precursors (pre-miRNAs) [95,96]. Then, the pre-miRNAs undergo subsequent cleavage by the DCL complex, forming a duplex structure [95].

Pre-miRNA in plants ranges from 49 to 900 nucleotides in length and undergoes processing by DCL1 or alternatively by DCL2, DCL3, and DCL4, forming a duplex with two laterally projecting 3′ nucleotides [95]. MiRNAs generally have 21 nucleotides, but the size varies depending on the DCL that performs the cleavage, with the distance between the RNase III and PAZ domains being suggested as a determinant of miRNA length [92,96]. The duplex is then methylated at the 3′ end by a methyltransferase, called HUA ENCHANCER1 (HEN1), to prevent further modification and degradation by exonucleases [97]. The HASTY protein (HST) transports the duplex to the cytoplasm [98], where one of the arms of the duplex is chosen and incorporated into a protein from the ARGONAUTA family (AGO) to form the RISC (RNA-Induced Silencing Complex). One of the domains of the AGO protein, called PIWI, has endonuclease activity and is capable of cleaving miRNA targets [98,99,100].

Elevated levels of CO_2_ and temperature can affect plant growth and development in general, but the signaling pathways that regulate these processes and which molecules are regulated by miRNAs under these conditions are still poorly understood. miRNAs can function in a complex regulatory network developed to deal with stressful conditions; several studies demonstrated that conserved miRNAs, such as miR160 and miR175, and new ones, had their expression altered by the elevation of temperature, which consequently resulted in the alteration of the expression of their genes target [36,39,101,102,103,104]; in addition, temperature rise can induce intron splicing of miRNAs [105].

In *A. thaliana*, the increase in temperature and CO_2_ were evaluated separately, and through small RNA sequencing, miRNAs whose expression is significantly altered in both situations were identified. Notably, almost all CO_2_-influenced miRNAs were inversely affected by elevated temperature. Furthermore, this study points out that the miR156/miR172 pathway, reported to act in the transition from vegetative to reproductive phase, probably has an important role in early flowering induced by high CO_2_ concentrations [105].

In sunflower (*Helianthus annuus*), a regulatory interaction between the transcription factor HaWRKY6 and miR396 was established. This miRNA, which normally acts in plant development through the regulation of GRF transcription factors, was activated for protection at high temperatures in sunflower (45 °C), a plant particularly well adapted to this type of stress. The identification of a young and divergent transcription factor under the negative control of a highly conserved regulatory element indicates that caution is needed when making inferences about the role of known miRNAs in non-model plants [106].

In the genus *Coffea*, several families of conserved and new microRNAs have been identified [42,43,45,107,108,109,110,111,112] in addition to the main proteins in the biosynthesis of sRNAs, such as DICER and Argonaute [44,113]. More recently, several types of non-coding RNAs were identified in *C. canephora*, in addition to miRNAs and their targets [114], and in *C. arabica* during floral development [46]. Hernández-Castellano et al. [115] identified some families of miRNAs in embryogenic callus; the data suggest a differential and cooperative role for miR535, miR164, miR2119, and miR157a during the initial stages of somatic embryogenesis in *C. canephora*, supporting their regulatory role in cellular totipotency of plants, which can bring benefits to genetic improvement programs. Regarding abiotic stress, a transcriptional profile of mRNAs and sRNAs in *C. arabica* roots has already been carried out in response to nitrogen lack [116]. In general, these data are important for the advancement of research with sRNAs in coffee plants; however, more studies are needed investigating the role of these molecules in adverse conditions such as high temperatures and high CO_2_ and their applicability as breeding tools.

SiRNAs, on the other hand, are generated from double-stranded RNAs (dsRNA) that can originate from different sources, such as RNAs transcribed from inverted repeats, natural cis-antisense transcription pairs, the action of RNA-dependent RNA polymerases (RDRs) that convert single-stranded RNA in dsRNA, the replication of RNA viruses, and regions of the genome rich in retroelements. DsRNA is cleaved into siRNAs of 21 to 24 nt by DCL proteins, and the size of siRNAs released depends on the specific catalytic activity of the respective DCL protein. DsRNA is usually cleaved by several DCL proteins, generating classes of siRNA with different sizes. Like miRNAs, siRNAs are loaded into RISC containing AGO protein that directs target regulation at either the post-transcriptional level or the transcriptional level through a pathway called RNA-directed DNA methylation (RdDM) [117]. 

DNA methylation is also an important epigenetic modification and plays a key role in regulating plant growth and development. In tobacco, the expression of methylated CycD3-1 and Nt-EXPA5 was found to be altered during heat stress [117]. In *Populus simonii*, the MIR393a, MIR156i, MIR167h, MIR396e, and MIR396g genes were methylated in CNG regions in heat-treated plants, while they were methylated in CG regions in cold-treated systems. These data demonstrate that methylation can regulate the expression of miRNAs under heat stress, further affecting the expression level of their targets, probably through the gene-silencing function of miRNAs [118].

The epigenetic marks are also being interpreted as part of the cell’s transcriptional memory in response to abiotic stress, so a deep comprehension of such mechanisms could assist in improving plant resilience. In agreement, a growing body of evidence suggests that the accumulation of signaling compounds and transcription factors (TFs) [118,119], together with epigenetic modification [120,121], play fundamental roles in the interplay between plant development and environmental signals. Very few examples are found in coffee; for example, Guedes et al. [122] studied the drought responses of tolerant and sensitive clones of *C. canephora* subjected to three drought cycles. The investigation of transcriptional memory to drought in the tolerant clone revealed genes related to ABA and a possible interaction between drought and biotic stress and memory genes. Furthermore, MYB proteins and miRNAs were found to modulate expression in the drought response. The drought-responsive genes identified in this work constitute valuable genomic resources to improve coffee cultivation and develop tolerant crops.

Finally, phasiRNAs constitute an important category of small RNAs in plants, but most of their functions are still poorly defined. The biogenesis of phasiRNAs occurs after cleavage of target mRNA or long non-coding RNA (lncRNA), typically (but not exclusively) by a 22-nucleotide miRNA [20]. After cleavage, the 5′ fragment of the target mRNA is rapidly degraded by a 3′→5′ exonucleolytic complex. The 3′ fragment is converted to double-stranded RNA (dsRNA) through the activity of (RDR6), which can be recruited by AGO1-RISC or AGO7-RISC and assisted by SUPPRESSOR OF GENE SILENCING3 (SGS3), which, in turn, can prevent degradation of the 3′ fragment of a 5′→3′ exoribonuclease. The resulting dsRNA is iteratively cleaved by a Dicer protein from the 5′ end of the “top” strand (derived from Pol II) containing the cleavage site, producing phasiRNA duplexes. The function of the Dicer family member DCL4 requires the assistance of a DOUBLE-STRANDED RNA BINDING FACTOR (DRB) protein to produce 21-nucleotide phasiRNAs. RISC containing phasiRNA subsequently interacts with target RNAs in a homology-dependent manner, such as with miRNAs [20].

Although phasiRNAs in plants are not yet fully elucidated as to their biological role, recent studies have shown that they play an important role in male fertility, such as in the development of anthers in maize (*Zea mays*) [123,124], wheat (*Triticum aestivum*), barley (*Hordeum vulgare*) [125], and rice (*Oryza sativa*) [126]; in addition, heat stress can negatively affect the reproduction of several species; recently, phasiRNAs have been found to be the most downregulated in response to heat stress in flax (*Linum usitatissimum*) reproductive tissues [126]. Recently, de Oliveira et al. [47] identified an increase in a specific group of phasiRNAs and the molecules involved in their biogenesis in the meiotic phase of coffee anthers. The meiotic phase of coffee anthers coincides with the G4 phase established by Moraes et al. [127], in which a latency stage of these buds is observed during the cold and dry periods in Brazil [68]. Furthermore, the pattern of accumulation of these molecules changes again after the rainy season and temperature increase, which demonstrates that these small molecules may be associated with the regulation of reproductive development in coffee plants in response to climate change. 

**Figure 1:**
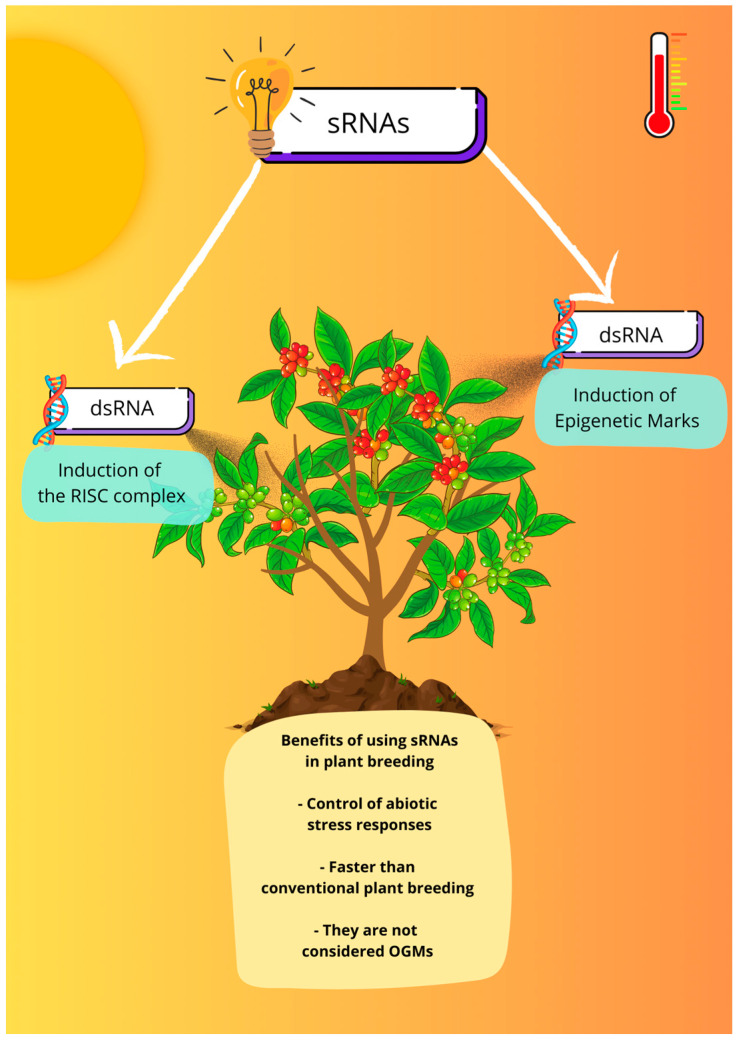
Small RNAs as a tool for plant breeding. Understanding the role of sRNAs in response to abiotic stress can help in the development of new products based on RNAi technology, such as the exogenous application of dsRNA that can induce the silencing of target genes or be used to induce epigenetic marks to stimulate the transcriptional memory.

As discussed above, sRNAs are molecules with great potential to be explored in the genetic improvement of coffee plants in the face of climate change. A good strategy for this is the use of RNA interference (RNAi) technology, which has already been extensively studied in plant disease control and plant protection strategies based on the knowledge that double-stranded RNA (dsRNA) molecules derived from plant or exogenous genes can silence essential or virulence genes in microbial pathogens and pests [128,129,130,131]. In this strategy, dsRNA induces the organism’s RISC silencing complex to silence target genes, as illustrated in Figure 1. These strategies are advantageous due to the highly specific, environmentally friendly, and malleable nature of dsRNAs [129].

## 6. Conclusions

Considering the negative impacts of climate change on crop production and the particular characteristics of species, extensive studies are needed to deeply comprehend the regulatory mechanism of plant development to direct breeding programs. This is particularly important because our diet largely depends on fruits, and reproductive development is strongly affected by temperature changes. Despite the identification of several sRNAs responsive to temperature and CO_2_ elevation, studies that explore these molecules in response to these changes in coffee plants are scarce. Here, we explored this gap, starting with a discussion on how the origin and phenology of the coffee tree are related to its low genetic variability. Then, we discussed how the physiological and molecular mechanisms respond to increased temperature and CO_2_ levels and are related to sRNA pathways. The role of sRNAs in different species and how they could be useful for crop breeding was also discussed, although it has been poorly explored in coffee plants. In conclusion, sRNAs and their related mechanisms must be better explored in crops due to their potential, which will allow for the development of new products based on RNAi technology that can be used exogenously, applied at specific periods of the development, and are better accepted by society than transgenics, in addition to overcoming the time barrier that is required in assisted breeding, especially for perennial species with complex phenology, such as coffee plants.

## Data Availability

Not applicable.
